# Genome Sequence of *Lactococcus lactis* subsp. *lactis* bv. diacetylactis LD61

**DOI:** 10.1128/genomeA.01176-13

**Published:** 2014-01-16

**Authors:** Hélène Falentin, Delphine Naquin, Valentin Loux, Frédérique Barloy-Hubler, Pascal Loubière, Sébastien Nouaille, Dominique Lavenier, Pascal Le Bourgeois, Patrice François, Jacques Schrenzel, David Hernandez, Sergine Even, Yves Le Loir

**Affiliations:** a INRA, UMR1253 Science et Technologie du Lait et de l'Œuf, Rennes, France; b AGROCAMPUS OUEST, UMR1253 Science et Technologie du Lait et de l'Œuf, Rennes, France; c INRIA/Irisa, GenScale Team, Campus de Beaulieu, Rennes, France; d INRA, MIG, INRA Domaine de Vilvert, Jouy-en-Josas, France; e CNRS, UMR6026 Interactions Cellulaires et Moléculaires, Campus de Beaulieu, Rennes, France; f Université de Toulouse, INSA, UPS, INP, LISBP, Toulouse, France; g INRA, UMR792 Ingénierie des Systèmes Biologiques et des Procédés, Toulouse, France; h CNRS, UMR5504, Toulouse, France; i Université de Toulouse, Université Paul Sabatier, Toulouse, France; j CNRS, Laboratoire de Microbiologie et de Genetique Moleculaires, Toulouse, France; k Genomic Research Laboratory, University of Geneva Hospitals (HUG), Geneva, Switzerland

## Abstract

*Lactococcus lactis* is widely used in the dairy industry. We report the draft genome sequence of *L. lactis* subsp. *lactis* bv. diacetylactis LD61, an industrial and extensively studied strain. In contrast to the closely related and plasmidless strain IL1403, LD61 contains 6 plasmids, and the genome sequence provides additional information related to adaptation to the dairy environment.

## GENOME ANNOUNCEMENT

*Lactococcus lactis* is widely used as a dairy starter in the production of fermented milk products, particularly cheese. *L. lactis* is the model lactic acid bacterium, and the genome of strain IL1403 was the first genome of a lactic acid bacterium (LAB) ever sequenced (1). *L. lactis* subsp. *lactis* IL1403 is a plasmidless dairy-derived strain, and three other sequenced *L. lactis* subsp. *lactis* strains were isolated from nondairy biotopes, i.e., mung bean sprouts for KF147 (2), vaginal flora for CV56 (3), and water of a kitchen sink for IO-1 (4). These three strains contain 1, 5, and 0 plasmids, respectively. These sequences provide interesting genomic data, allowing for the identification of mechanisms of adaptation to peculiar niches, but they do not allow for a fine-tuned analysis of genomic diversity within genetically related clusters. Yet, an integrated approach toward *L. lactis* subsp. *lactis* provided evidence of intrasubspecies genetic diversity (5).

*L. lactis* subsp. *lactis* bv. diacetylactis strain LD61 was isolated from a starter culture and has a dairy phenotype. It contains 6 plasmids, the sizes of which were estimated to be 4.5, 6.5, 9, 11, 50, and 55 kb, and which carry important components for sustaining growth in milk (citrate permease operon, lactose operon, and cell wall protease). It has been extensively used in recent works regarding gene expression and the inhibition of *Staphylococcus aureus* virulence expression under various growth conditions (6–8). The whole-genome sequencing of *L. lactis* subsp. *lactis* LD61 reveals genomic diversity of *L. lactis* subsp. *lactis* involved in the different capabilities observed among dairy strains.

The LD61 whole genome was sequenced by using an Illumina HiSeq 2000 (Fasteris, Geneva, Switzerland). Base calling was performed using the HiSeq Control software version 1.4.8. After barcode selection, 9.2 million paired-end reads of 100 bases in length were obtained. The sequence reads were *de novo* assembled using the Edena assembler version 3.131028 (9, 10). Assembly resulted in 132 contigs (sum, 2.60 Mbp; N_50_, 48.2 Kbp; max, 252.7 Kbp; min, 200 bp) with a G+C content of 36.4%. A total of 2,601 coding sequences were detected by using the NCBI Prokaryotic Genomes Automatic Annotation Pipeline (PGAAP) (11). Over 45% of the genes were assigned to specific subsystem categories by RAST (12). A more detailed analysis of this genome and comparative analyses with other *L. lactis* subsp. *lactis* genomes will provide further insight into the genomic differences and genome evolution within this species.

### Nucleotide sequence accession numbers.

This whole-genome shotgun project has been deposited at DDBJ/EMBL/GenBank under the accession no. AXZK00000000. The version described in this paper is version AXZK01000000.
